# DNA methylation markers predict recurrence-free interval in triple-negative breast cancer

**DOI:** 10.1038/s41523-020-0145-3

**Published:** 2020-01-31

**Authors:** Mary Jo Fackler, Soonweng Cho, Leslie Cope, Edward Gabrielson, Kala Visvanathan, Kathleen Wilsbach, Danielle Meir-Levi, Charles F. Lynch, Jeffrey Marks, Joseph Geradts, Meredith M. Regan, Giuseppe Viale, Antonio C. Wolff, Saraswati Sukumar, Christopher B. Umbricht

**Affiliations:** 1 Department of Oncology, Johns Hopkins University School of Medicine, Baltimore, MD 21205 USA; 2 Department of Surgery, Johns Hopkins University School of Medicine, Baltimore, MD 21205 USA; 3 Department of Pathology, Johns Hopkins University School of Medicine, Baltimore, MD 21205 USA; 4 Department of Epidemiology, Johns Hopkins University School of Public Health, Baltimore, MD 21205 USA; 5 SEER Tissue Repository Program, State Health Registry of Iowa, Iowa City, IA 52242 USA; 6 Department of Surgery, Duke University Medical Center, Durham, NC 27710 USA; 7 Department of Pathology, Duke University Medical Center, Durham, NC 27710 USA; 8 Department of Population Sciences, City of Hope National Medical Center, Duarte, CA 91010 USA; 9 IBCSG Statistical Center, Department of Biostatistics and Computational Biology, Dana-Farber Cancer Institute, Harvard Medical School, Boston, MA 02215 USA; 10 Department of Pathology, IEO European Institute of Oncology IRCCS and University of Milan, Milan, Italy

**Keywords:** Prognostic markers, Breast cancer

## Abstract

We lack tools to risk-stratify triple-negative breast cancer (TNBC). Our goal was to develop molecular tools to predict disease recurrence. Methylation array analysis was performed on 110 samples treated by locoregional therapy obtained from institutional cohorts. Discovered marker sets were then tested by Kaplan−Meier analyses in a prospectively collected TNBC cohort of 49 samples from the no-chemotherapy arms of IBCSG trials VIII and IX, and by logistic regression in a chemotherapy-treated cohort of 121 TNBCs from combined IBCSG trials and institutional repositories. High methylation was associated with shorter recurrence-free interval in the no-chemotherapy arm of the IBCSG studies, as well as in the chemotherapy-treated patients within the combined institutional and IBCSG chemotherapy cohorts (100 marker panel, *p* = 0.002; 30 marker panel, *p* = 0.05). Chromosome 19 sites were enriched among these loci. In conclusion, our hypermethylation signatures identify increased recurrence risk independent of whether patients receive chemotherapy.

## Introduction

Triple-negative breast cancers (TNBC) lack expression of the estrogen (ER), progesterone (PR) and HER2 receptors. The TNBC subtype represents ~15% of all breast cancers and is more aggressive than other subtypes of breast cancer, with poorer prognosis. In recent studies, the 5-year overall survival was 60%^1^ and the breast cancer-specific survival was 75%.^2^ The absence of receptors renders TNBCs unresponsive to the targeted hormonal and anti-HER2 therapies that are used in other breast cancers. Unfortunately, none of the most widely used gene expression profiling tests, the 21-gene Oncotype DX, the 70-gene Mammaprint, or the PAM50, has clinical utility in patients with TNBC.^3,4^

On the other hand, combined data from several National Adjuvant Surgical Breast and Bowel Project (NSABP) trials show that in early disease, 80% of patients with ER-negative tumors measuring 1 cm or less (T1a-b) treated with locoregional therapy alone remained recurrence free at 8 years.^5^ Identification of the 20% of early-stage TNBC tumors with more aggressive behavior would provide two benefits: (1) patients with less aggressive disease could be managed more conservatively, and, (2) patients known to have more aggressive disease could be selected for more intensive and/or novel therapies at earlier stages of disease.

Similar to gene expression, global patterns of DNA methylation in cancer can define distinct breast cancer subtypes^6^ with important prognostic implications.^7^ Gene promoter methylation is an important silencing mechanism for tumor suppressors and associated regulatory genes,^8^ and dysregulation of DNA methylation is an early hallmark of cancer and has been well-documented in in situ neoplasia as well as invasive and metastatic carcinoma.^9,10^ It may be possible to exploit these features of the disease to risk-stratify TNBC tumors.

**Fig. 1:**
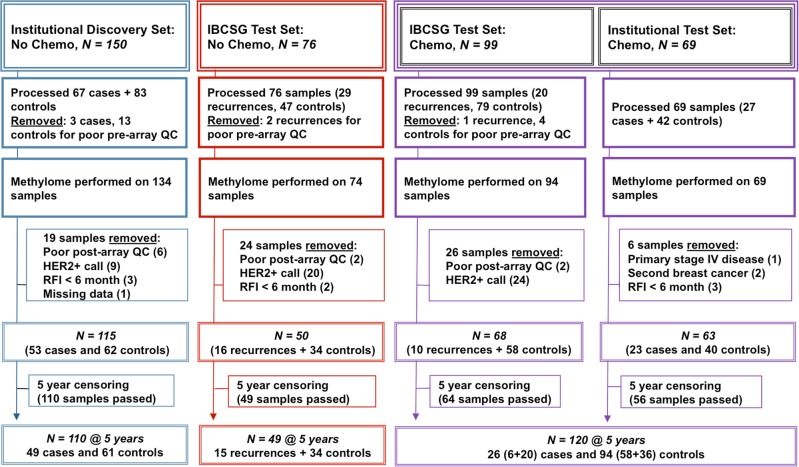
Consort diagram showing sample sources and processing scheme for identification of markers. Primary TNBC cancer samples from the Discovery cohort (*N* = 110) (not given chemotherapy) were evaluated to identify DNA methylation markers high in recurring and low in nonrecurring cancers. Candidate markers were then tested in the IBCSG No Chemo Test cohort (not given chemotherapy) (*N* = 49). Also evaluated was the combined IBCSG and Institutional Chemo cohort (*N* = 120) obtained from patients who received chemotherapy. Cases that recurred after 5 years and controls with less than 5 years of follow-up were excluded (5-year censoring).

The objectives of this study were to develop prognostic tools for TNBC that can distinguish breast cancers with a favorable natural history vs. those with a high risk of recurrence. We hypothesized that methylated gene markers present in primary TNBC will help identify: (1) the majority of early-stage patients (60%) whose cancer will not recur after locoregional therapy alone, and therefore may not benefit from chemotherapy, and (2) those who will draw benefit from chemotherapy (20%), and (3) those who will not benefit (20%) and therefore need alternate treatment approaches. Towards this goal we performed a genome-wide search for DNA methylation markers using the available archival tumor samples of node-negative TNBC from three institutions (Johns Hopkins University, University of Iowa, Duke University) as well as available samples from TNBC patients enrolled in International Breast Cancer Study Group (IBCSG) Trials VIII and IX.^11^ We required a minimum follow-up of 5 years post diagnosis, and allowed both chemotherapy-treated and -untreated patients with preplanned stratification in this exploratory phase of assay development (see consort diagram, Fig. 1).

## Results

### Quality control (Q/C)

The overall Discovery strategy used to identify CpG loci associated with recurrence is shown in Supplementary Fig. S1. The technical performance for the array was assessed within GenomeStudio for each sample by determining the percentage of detected CpG probes passing quality control (*p* ≤ 0.01) among the 485,577 array probes (Supplementary Fig. S2). Mean call rates for the study sample groups, Institutional NoChemo, Institutional Chemo, IBCSG (NoChemo & Chemo), and Normal Breast sample set, were 95%, 96%, 87% and 98%, respectively. Samples deemed to be outliers (≥2.5 SD from the mean) were removed from the study (Supplementary Fig. S2).

The key clinical parameters for the resulting three study cohorts (Institutional NoChemo, Discovery Set, *N* = 115; IBCSG NoChemo, Test Set1, *N* = 50; Combined Chemo, Test Set2, *N* = 131) are summarized in Table 1. As expected, NoChemo and Chemo cohorts differed significantly in age, tumor size, and use of radiotherapy, while tumor grade did not.

**Table 1:** Characteristics of the primary TNBC breast cancer patients in the study.

Characteristics	Discovery set No chemotherapy	IBCSG test set No Chemotherapy	IBCSG/JHU test set Chemotherapy	
Total	*N* = 115	*N* = 50	*N* = 68 + 63	
Cases (with recurrences) (*n*)	53		10 + 23	
Controls (no recurrences) (*n*)	62		58 + 40	
Recurrence-free interval (median months)		125		
Age (median)	63	54	50	*F* = 34.87, *p* < 0.00001
AJCC stage				*χ*^2^ = 41.51, *p* < 0.00001
I	29	7	13 + 19	
II	48	42	55 + 31	
III	2	0	0 + 13	
NA	36	1	0	
Tumor stage				*χ*^2^ = 13.84, *p* = 0.0078
1	14	26	44	
2	35	19	77	
3	0	5	10	
NA	65	1	0	
Grade				*χ*^2^ = 3.60, *p* = 0.46
1	0	0	2	
2	9	19	18	
3	41	78	111	
NA	18	0	0	
Radiation therapy (*n*)	33	19	64	*χ*^2^ = 10.48, *p* = 0.0053

### Exploratory data analysis

#### Principal component analysis (PCA)

Two principal component analyses (PCA), incorporating all 460,772 array probes (Supplementary Fig. S3a) and 59,581 cancer-specific probes (Supplementary Fig. S3b) respectively, were performed on the methylation data of the Institutional NoChemo (Discovery) dataset to visualize similarities between samples. While the first three components showed clear separation between tumor and normal samples, recurrent and nonrecurrent tumors largely overlapped, indicating most of the array probes were similarly methylated between recurrence groups.

### High differential methylation of candidate CpG loci detected in recurrent tumors vs. nonrecurrent tumors in the Discovery set of patients receiving no chemotherapy

Starting with the 59,581 cancer-specific CpG probes, we ranked candidate recurrence-associated probes from highest to lowest based on the MeanRatio in Partek Genomic Suites, calculated by dividing the average methylation beta value within the recurrent (R) group by that of the nonrecurrent (NR) group (MeanRatio = R/NR), selecting the 100 most highly ranked probes for further analysis (Table S1). Clinical utility depends on both intensity and frequency of methylation of a marker in individual cancer and normal tissues. Therefore, we inspected the distributions of methylation levels for each of the 100 probes in both tumor and normal breast samples. A total of 30 probes having *β* < 0.01 in all normal breast samples, as well as *β* > 0.015 in at least 4 of 110 tumors, were identified as particularly promising candidates (Table S1). Individual CpG methylation levels among genes in the panel were added together within a sample to obtain a cumulative methylation index (CMI) for each subject.

**Fig. 2:**
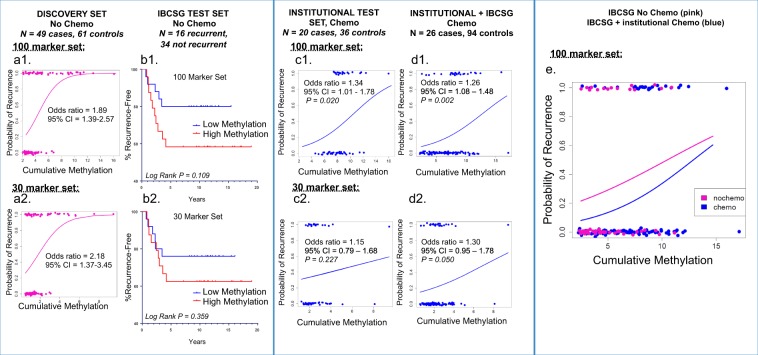
Logistic regression plots demonstrate the relationship between the probability of recurrence vs. high cumulative methylation levels of the panel of 100 CpG markers, and 30 CpG marker subset, identified using the Discovery cohort. Sample cumulative methylation is defined for the marker panel as the sum of beta methylation (*β* value, the level of CpG locus methylation, low to high ranging from 0 to 1, respectively for each marker in the panel). Cases (recurrences, upper *x*-axis) and controls (no recurrences, lower *x*-axis) are shown as colored circles. The association between methylation level and probability of recurrence (*y* axis), at 1.0 and 0.0 probabilities respectively, is demonstrated in plots (**a**, **c**–**e**). The likelihood of recurrence increased in proportion to the level of cumulative methylation, as seen in cohorts from the Discovery (a1–a2, No Chemo), Institutional (c1−c2, Chemo; *p* = 0.020, 100 marker panel), and Institutional + IBCSG (d1−d2, Combined Chemo; *p* = 0.002, 100 marker and *p* *=* 0.050, 30 marker sets), using each group’s median methylation as the threshold value. The overlay of regression plots (E1) derived from the IBCSG No Chemo cohort (pink, upper line) and the IBCSG/Institutional Combined Chemo cohort (blue) shows similar upward slopes suggesting that the association between high methylation and increased risk is independent of chemotherapy. The Kaplan−Meier plot of the IBCSG Test Set (b1−b2, No Chemo) displays the relationship between high methylation (red, lower line) and shorter recurrence-free survival, compared to low methylation (blue).

The relationship between CMI and recurrence in the Discovery cohort, modeled using logistic regression, is shown in Fig. 2a, for both the 100 and the 30 CpG probe panels. Fitted logistic regression curves, describing the estimated probability of recurrence for each methylation level, showed a strong association of higher cumulative methylation levels of the gene marker panel with recurrence (Fig. 2a1, Supplementary Tables S1 and S2).

### High methylation is associated with increased probability of tumor recurrence in patients who do not receive chemotherapy

To determine if the differentially methylated panels of 100 and 30 markers could identify a subgroup of TNBC patients who had good outcomes without receiving chemotherapy, we evaluated the nonchemotherapy arms of IBCSG Trials VIII & IX. The Kaplan−Meier plot generated by comparing samples with high vs. low methylation relative to the median showed that patient with high tumor methylation had shorter recurrence-free intervals (RFI),^12^ although data did not reach statistical significance in this cohort with only 16 recurrences (Fig. 2b1, b2, Supplementary Fig. S4).

### High methylation is associated with increased probability of tumor recurrence in patients who receive chemotherapy

Our next step was to determine whether the same markers could stratify TNBC patients who received chemotherapy, according to risk of recurrence. For this purpose, we combined an institutional chemo-treated set (*N* *=* 56, 20 cases and 36 controls) with data from the IBCSG chemo-treated arm (*N* *=* 64, 6 cases and 58 controls), applying the same definition of case and control according to 5-year, recurrence-free survival to both sets. Logistic regression was used to model the relationship between DNA methylation level and disease status at 5 years. We observed a significant association between high methylation and a recurrence for the 100 marker panel (OR = 1.26, *p* *=* 0.004), while the association was weaker for the 30 marker panel (OR = 1.30, *p* = 0.10) (Fig. 2d1, d2, Supplementary Fig. S4). Odds ratios were similar for the institutional samples, evaluated separately (100 marker panel: OR = 1.34, *p* = 0.02; 30 marker panel: OR = 1.15, *p* = 0.227) (Fig. 2c1, c2, Supplementary Fig. S4). With only six cases, IBCSG chemo-treated samples were not evaluated separately. Finally, when analyzed as single markers, 14 of the 30 CpG loci showed significant associations between high DNA methylation and recurrence (Supplementary Table S2) for the combined chemo-treated group. Incidental to these findings, we observed that the number of CpG loci probes localizing to Chromosome 19 was enriched in our panels compared to the array as a whole. Only 5.3% of the array probes map to Chromosome 19, but the recurrence panels of 100 and of 30 markers were enriched to 15 and 37% Chr. 19 probes, respectively (Supplementary Table S3).

Finally, all test samples in the NoChemo and Chemo groups were evaluated together using a single median methylation array value (*β* = 6.469) to define high vs. low methylation (Fig. 2e). In this analysis, high methylation was associated with worse outcome regardless of adjuvant chemotherapy, and chemotherapy improved outcome regardless of methylation levels. The observed narrowing of the curves at high cumulative methylation values, where probability of progression was highest, may suggest a better response to chemotherapy in low methylation cancers, but there are too few data points supporting the model in the high methylation range to draw confident conclusions.

### Cross-platform technical array validation

To ensure accuracy of our array data obtained from archival tissue, we used an alternative assay, QM-MSP, to measure DNA methylation levels for a subset of the markers. Using 42 of the 50 IBCSG NoChemo samples, including 12 recurrences, we compared results between QM-MSP and the array for 9 markers that had frequent array hypermethylation in both the Discovery Set and IBCSG NoChemo Test Set. The QM-MSP results paralleled that of the methylation array, with a Spearman correlation coefficient of *r* = 0.495 (*p* = 0.0009) (Supplementary Fig. S5).

## Discussion

The purpose of this study was to investigate whether DNA methylation patterns might stratify TNBCs into clinically actionable risk categories using available clinical samples of convenience for which long-term outcomes were well documented. The primary analysis included TNBC patients who received only locoregional therapy, after which the findings were extended to include patients treated by chemotherapy as well.

We identified a set of 100 candidate CpG marker sites, as well as a more selected set of 30 marker sites, in which higher levels of methylation were associated with greater probability of 5-year recurrence following locoregional therapy. In both panels, high levels of methylation were associated with worse outcome in independent samples from patients enrolled in IBCSG Trials VIII and IX, who received locoregional treatment but no chemotherapy, although results did not reach statistical significance.

We further tested our candidate markers in samples from patients who received chemotherapy as well as locoregional treatment. Like the locoregionally treated samples, these included patients from the chemo-treated arms of IBCSG Trials VIII and IX, as well as institutional samples of convenience. The samples were combined and analyzed as one case−control cohort using logistic regression to model the relationship between DNA methylation and 5-year recurrence. Again, higher levels of methylation were associated with an increased probability of recurrence, with the result achieving statistical significance this time.

The 30 CpG marker panel appeared to have less discriminatory power compared to the 100 marker panel, which may be a reflection of the well-documented heterogeneity present in TNBC,^13^ indicating that adequately characterizing TNBC will require broad molecular signatures. The 30 CpG loci were enriched in genes that demonstrate DNA binding and transcription regulatory functions, and Chromosome 19-specific probes were enriched among the 100 and 30 CpG marker sets compared to the array as a whole (see Supplementary Table S3).

Finally, we fit a multivariate logistic regression model, including both chemo- and locoregionally treated samples, to answer our primary question of whether the potential benefits of chemotherapy varied by methylation level. We found that patients consistently benefit from chemotherapy across the entire range of methylation levels, results that support the current, standard practice of providing adjuvant chemotherapy to all TNBC patients.

The strengths of our study include assembling a set of now rare, TNBC cases treated without chemotherapy, the availability of robust long-term outcome data, as well as samples from mature international randomized controlled trials, and the comprehensive, genome-wide analysis of DNA methylation in this study. Limitations include the retrospective analysis of a heterogeneous set of samples from several sources, necessary to complement the small number of recurrences observed in IBCSG trials VIII and IX, and the inability to adjust for clinicopathologic covariates. It was therefore encouraging to see that results were comparable across our diverse cohorts, including in the chemotherapy arms of our institutional samples and IBCSG trial samples.

In summary, our finding of a repeated association between methylation patterns and long-term outcome suggest that further exploration of their potential to better risk-stratify patients in well-characterized TNBC datasets may be warranted. We have identified patterns of methylation that are associated with prognosis among patients with TNBC, although they are at present not predictive of response to local-regional or systemic therapy. While these findings cannot be exploited to improve clinical decision making today, our results, and the underlying data, collected on an increasingly rare group of TNBC patients treated with locoregional therapy alone, are a valuable contribution to ongoing research on the development, progression and ultimate treatment of TNBC.

## Methods

### Informed consent

All procedures performed in studies involving human participants were in accordance with the ethical standards of the institutional and/or national research committee and with the 1964 Helsinki Declaration and its later amendments or comparable ethical standards.

Women enrolling in the study signed an informed consent approved by the Institutional Review Boards of participating institutions (Johns Hopkins University, Duke University, University of Iowa). For IBCSG study samples, institutional review boards reviewed and approved the protocols, and informed consent was required according to the criteria established within the individual countries.

### Sample selection

This study was designed and reported in accordance with REMARK guidelines.^14^ Our experiment required at least 75 cases and 75 controls in the discovery phase as well as at least 83 trial samples for validation. The discovery phase had to provide adequate power for candidate marker discovery and the validation phase had to permit precise estimation of marker performance. Simulations show that with 75 samples per group, and the false discovery rate (FDR) controlled at no more than 10%, we expect to identify the majority of sites (62%) differentially methylated at a moderate effect size of 0.75 SD and virtually all (98%) of those with an effect size of 1.0 SD. Moreover, the 95% lower confidence bound on sensitivity/specificity will be within 0.1 of the estimate. With 83 samples, the validation study would be powered to detect a hazard ratio of 2.3 between patients with high- and low-risk methylation profiles, corresponding to 10-year survival of approximately 60% and 80% respectively, based on long-term survival rates for locoregionally and systemically treated TNBC. Moreover, the 95% lower confidence bound on sensitivity/specificity will be within 0.1 of the estimate. The number of available TNBC samples meeting our selection criteria for patients receiving only locoregional therapy (see Fig. 1) was, however, very limited after the publication of studies documenting the benefits of adjuvant chemotherapy in TNBC. While the ultimately available IBCSG samples included fewer recurrence events than specified in our design, we continued because of the need for improving our understanding of TNBC and because material from locoregionally treated TNBC tumors would only become more difficult to obtain in the future.

The consort diagram shows our sample selection and quality control (Q/C) process (Fig. 1). Formalin-fixed paraffin-embedded (FFPE) tissues of node-negative primary TNBC, stages 1–3, were obtained after review of pathology tissue archives and clinical reports from collaborating laboratories at Johns Hopkins University (JHU), Duke University Medical Center, and the State Health Registry of Iowa, with approval of the respective Institutional Review Boards, as institutional cases and controls. The institutional tissue blocks were collected over a 25-year period (1985–2009). Samples were also accessed from residual materials from The International Breast Cancer Study Group (IBCSG) Randomized Clinical Trials VIII and IX,^11^ after approval of the International Breast Cancer Study Group Biological Protocols Working Group (Table 1).

Many of the tissue samples obtained for this study predate the availability of standardized assays for HER2 status, and the definition of hormone receptor status changed over time as well. For all IBCSG samples, we relied on the centralized ER and HER2 assessment available from IBCSG.^11^ For institutional samples, we accepted the original classifications provided in the official pathology reports of the respective institutions. For samples predating routine HER2 assessment, we performed a transcriptome analysis using the Illumina DASL arrays^15^ to detect HER2 transcript expression. HER2 positivity was assigned using the Q3 + 1.5*IQR (inter-quartile range) rule identifying HER2 outliers.

Institutional cases were defined as those with local, regional or distant recurrence, which occurred between 6 months and 5 years after initial diagnosis but excluded contralateral breast cancer. Controls were those with no recurrence, with a minimum follow-up of 5 years. Cases that recurred after 5 years and controls with less than 5 years of follow-up were excluded (5-year censoring). The same criteria were applied to the IBCSG samples. Institutional controls were matched to cases by age and date of diagnosis within 5 years, AJCC (eighth edition) stage, and radiotherapy as first course therapy. Samples from patients that received no chemotherapy (NoChemo) were analyzed separately from those receiving chemotherapy (Chemo), but specific chemotherapy regimens (CMF for IBCSG, CMF and Anthracycline/Taxane regimens for institutional samples) were not further stratified for the institutional sets. Samples with incomplete data were removed from the analysis. Similarly, use of hormonal agents was not used to stratify the cohorts since they have not been shown to affect outcome in TNBC. Additionally, five FFPE samples of normal breast tissue obtained from JHUSOM Surgical Pathology were processed in parallel.

This approach resulted in four sets of samples (see Fig. 1): the Institutional NoChemo set (*N* = 150, with 110 passing all array Q/C and 5-year censoring), used for initial marker discovery; the IBCSG NoChemo set (*N* = 76, 49 passing Q/C and 5-year censoring); the IBCSG Chemo set (*N* = 99, 64 passing Q/C and 5-year censoring); and the Institutional Chemo set (*N* = 69, 56 passing Q/C and 5-year censoring).

Candidate methylation markers identified in the NoChemo discovery cohort were then independently validated on the IBCSG NoChemo dataset, and separately on a Chemo dataset combining available Institutional and IBCSG Chemo samples (*N* = 64 + 56 = 120).

### Genomic DNA extraction, sodium bisulfite conversion, and template quality control (Q/C)

All study samples were assigned internal codes and all laboratory analyses were performed in a blinded fashion with regard to sample outcomes. FFPE tumor tissue sections (Institutional samples, two 10 μm sections with >50% tumor, macrodissected as necessary) or one tumor-targeted core of FFPE (IBCSG samples, 0.6 mm × 2 mm) were deparaffinized with xylene, digested for 24 h at 56 °C with 10 mM Tris pH 8, 150 mM NaCl, 2 mM ethylenediaminetetraacetic acid (EDTA), 0.5% sodium dodecyl sulfate (SDS) containing 4 mg proteinase K, and heated to 90 °C for 10 min. The EZ DNA Methylation kit (D5001; ZymoResearch) was used for bisulfite conversion. Bisulfite-treated genomic DNA was restored (375 ng in 10 µl; Infinium HD FFPE DNA Restore Kit; WG-321-1002, Illumina, Inc.) and arrayed using the Illumina Infinium HumanMethylation450K BeadChip Kit (WG-314-1003) in the JHU DNA Microarray Core. Prior to hybridization, DNA quality was verified by Nanodrop, and samples with less than 300 ng DNA were rejected. After samples were arrayed, the amount of hybridized DNA on the array was evaluated by comparing the total signal of methylated and unmethylated DNA to background levels at each CpG locus. High, locus-specific *detection p values* (*p* > 0.01) indicate low DNA levels, poor quality of DNA, or otherwise poor probe performance. Samples were rejected that had too many poorly performing probes (>2.5 standard deviations below the mean % probes passing QC; see Fig. S1).

### Quantitative multiplex methylation-specific PCR (QM-MSP)

QM-MSP^16^ primer/probe sets were designed to hybridize to a region overlapping or within 100 bp of the target sequence for the 50 bp array probe of interest. Individual gene methylation (M) is calculated as $\% {\mathrm{M}}\,{\mathrm{gene}} = \frac{{\left( {\# {\mathrm{methylated}}\,{\mathrm{copies}}} \right)}}{{(\# \,{\mathrm {methylated}} \,+\, {\mathrm {unmethylated}}\,{\mathrm {copies}})}}[100]$. Cumulative methylation index (CMI) = sum of % M for all genes.^16^

### Statistical analysis

Raw array file data were imported into Illumina GenomeStudio v2011.1 (Illumina, Inc, San Diego, CA) without normalization. Probe hybridization and quality control were assessed within GenomeStudio, as was the level of CpG locus methylation, calculated as a *β* value, low to high ranging from 0 to 1, respectively. Distributions of methylation levels were visualized using histograms. Data were then imported into the Partek Genomics Suite (Partek, Inc, St. Louis, MO) and analyzed using standard Partek functions as well as GraphPad Prism 5.0 (GraphPad Software, La Jolla, CA) tools. A set of 59,581 CpG sites with a tumor/normal mean *β* value ratio ≥1.5 were designated as cancer-specific and evaluated separately in some of the subsequent analyses. The selection of high tumor/normal *β* value ratios minimizes the contribution of benign tissue and variable sample purity to the cumulative methylation index.

Log rank (Mantel−Cox) tests were used to evaluate associations between DNA methylation levels and recurrence in IBCSG trial data. Because our institutional, case−control cohorts are selected for outcome, they do not reflect the natural history of disease and estimation of hazards would be inappropriate. Accordingly, logistic regression analysis was used to analyze institutional, and mixed, IBCSG/Institutional datasets. For the mixed analysis, the trial data were converted to cases vs. controls by classifying them according to recurrence status at the 5-year mark. Models were fit without adjustment for clinical covariates, in consideration of the risk of over-fitting a complex model on a moderately sized dataset. We also note that adjuvant chemo is the current standard of care for TNBC under a very broad spectrum of clinical parameters, suggesting that clinical covariates we can consider have limited use in this situation. Kaplan−Meier curves, logistic regression curves and boxplots, and Mann−Whitney tests were used to visualize associations and compare recurrence groups. Selected markers were evaluated by Quantitative Multiplex Methylation-Specific PCR (QM-MSP) to verify the methylation measures obtained by array, and results were compared using Spearman correlation. All tests were two-tailed and *p* < 0.05 was considered significant.

### Reporting summary

Further information on research design is available in the Nature Research Reporting Summary linked to this article.

## Supplementary information

Supplementary Figures and Tables.

Reporting Summary Checklist

## Data Availability

The data that support the findings of this study that were derived from Institutional patient samples are available through the GEO repository at https://identifiers.org/geo:GSE141441 (2019). All data derived from IBCSG study samples have been deposited with the IBCSG Statistical Center. Access to IBCSG-derived datasets are contingent upon IBSCG review and approval, for which a Data Use Agreement may be required. To request access to data, contact the IBCSG Statistical Center (stat_center@ibcsg.org). The IBCSG requires notification and opportunity to review before any public dissemination of results. The data generated and analyzed during this study are described in the following data record: 10.6084/m9.figshare.11378790.^17^
