# Supplementary material for: DNA methylation markers predict recurrence-free interval in triple-negative breast cancer
Source: NPJ Breast Cancer. 2020 Jan 31;6:3. doi: 10.1038/s41523-020-0145-3 (PMC6994477; doi:10.1038/s41523-020-0145-3)
Supplement: Supplementary file 1 — Supplementary Figures and Tables. [file 41523_2020_145_MOESM1_ESM.pdf]

Supplementary Figures & Tables

Supplementary Figure 1: Percentage of array probes passing QC. Individual samples were evaluated for overall performance. Shown is the percentage of probes with Detected CpG $p \leq 0.01$ (high probe performance), data extracted from the Sample Table within GenomeStudio. Samples with low performance (> 2.5 SD below the mean), shown below the horizontal line, were removed. The table at right of each graph shows the descriptive statistics of the sample set.

Supplementary Figure 2: Marker Selection Strategy. Recurrence-associated probes were identified using the Discovery Set of TNBC tumors (T) from patients who received loco-regional therapy only (IBCSG Test Set #1). Normal breast tissues (N = 5; NBr) served as controls. Markers were filtered in a step-wise progression, as indicated.

The final marker set of 100 and a subset consisting of 30 markers identified based on the Discovery sample set only.

Supplementary Figure 3: Principal Component Analysis (PCA) was performed to visually inspect the methylation status of CpG loci probes among recurrent (blue ellipsoid) tumors versus non-recurrent tumors (brown ellipsoid). Each dot is an individual sample (Normal Breast samples, green, n = 5; tumor, red, n = 115) and represents the average methylation status across the CpG loci. A. 460,772/485,577 total CpG probes (detection p-value <0.01). B. 59,581 cancer-specific probes (mean ratio Tumor/Normal samples ≥ 1.5), 12% of total CpG probes.

The scale for the axes is the same as in A). X- and Y- axes are re-scaling to enable closer visual inspection of the ellipsoids.

Supplementary Figure 4: Hypermethylation of 100 and 30 Marker Sets in Recurrent versus Non-recurrent Breast Cancers. The sum of beta array methylation for each 100 marker set and the 30 marker subset was calculated and displayed as box/whiskers plots (minimum to

maximum). For the test cohorts, the Mann-Whitney test was used to compare methylation levels among primary breast cancers that were Recurrent (R) versus Non-recurrent (NR).

Supplementary Figure 5: Cross-Platform Technical Validation of the Methylation Array. Quantitative Multiplex Methylation Specific PCR (QM-MSP) was performed using a 9 CpG markers chosen from the 30 marker set according to their overall frequent hypermethylation in both the Discovery and IBCSG NoChemo Test arrays. Spearman Correlation shows strong correlation between array and QM-MSP methods.

Supplementary Table 1. 100 CpG Recurrence Markers Selected in the Discovery Cohort.

Supplementary Table 2. Chromosomal Localization of Methylated CpG Loci in the Combined Chemo Group. Logistic regression p-value is based on median methylation for each individual CpG locus of the 30 marker set in the combined IBCSG + Institutional Chemo cohort (26 cases and 95 controls). Correction for multiple testing was not performed.

Supplementary Table 3. Enrichment of CpG Loci Located on Chromosome 19.

Supplementary Figure 1.

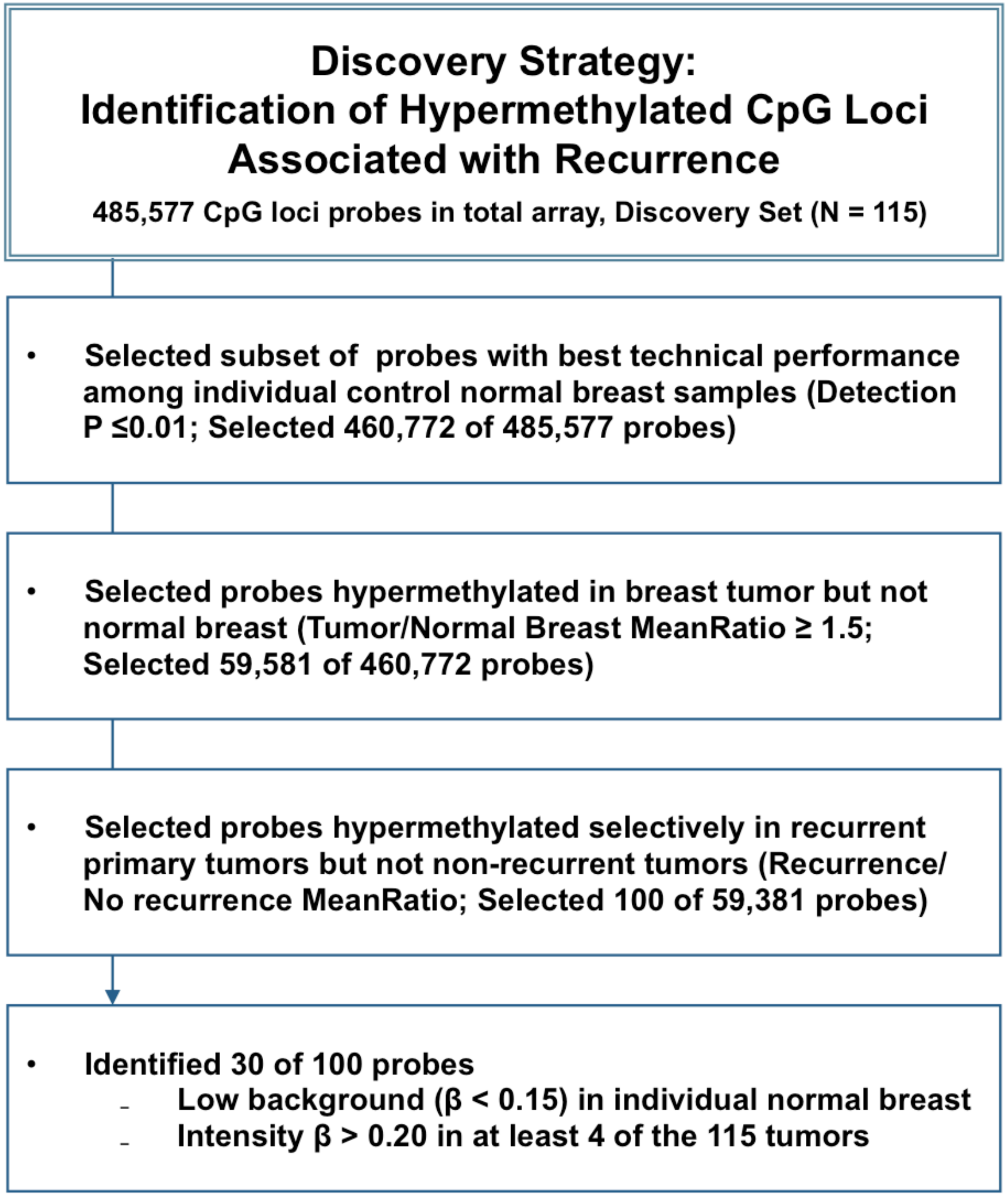

Supplementary Figure 2.

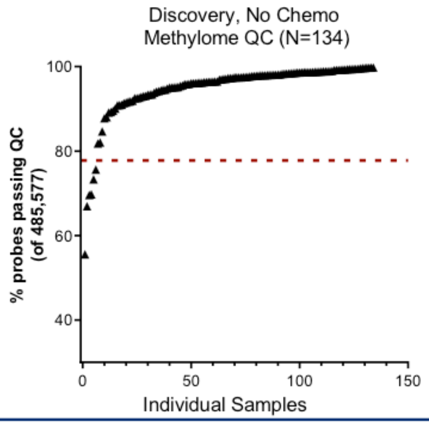

	% Probes Passing QC (p< 0.01)
Number of samples	134
Minimum	55.54
25% Percentile	94.01
Median	97.28
75% Percentile	98.64
Maximum	99.79
Mean	94.89
Std. Deviation	6.841
Std. Error	0.591
Lower 95% CI of mean	93.72
Upper 95% CI of mean	96.06

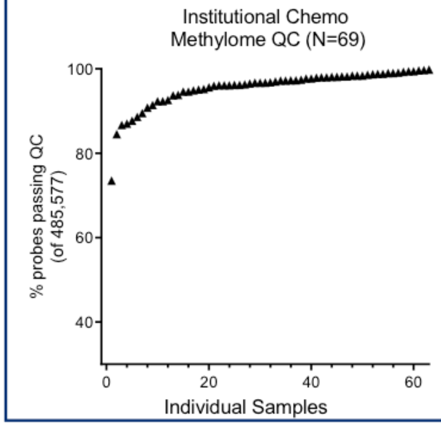

	% Probes Passing QC (p< 0.01)
Number of samples	69
Minimum	73.50
25% Percentile	94.60
Median	96.80
75% Percentile	98.40
Maximum	99.80
Mean	95.56
Std. Deviation	4.51
Std. Error	0.568
Lower 95% CI of mean	94.43
Upper 95% CI of mean	96.70

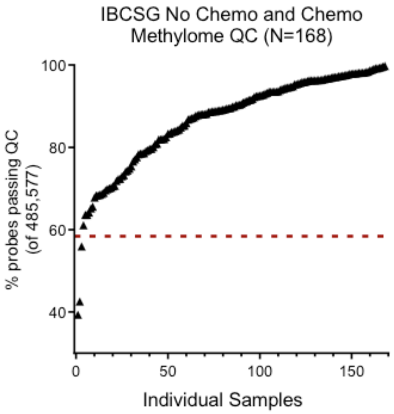

	% Probes Passing QC (p< 0.01)
Number of samples	168
Minimum	39.32
25% Percentile	80.25
Median	89.71
75% Percentile	96.22
Maximum	99.78
Mean	86.86
Std. Deviation	11.38
Std. Error	0.878
Lower 95% CI of mean	85.12
Upper 95% CI of mean	88.59

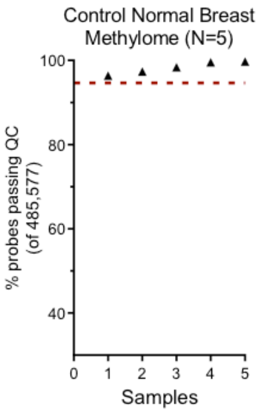

	% Probes Passing QC (p< 0.01)
Number of values	5
Minimum	96.33
25% Percentile	96.84
Median	98.35
75% Percentile	99.63
Maximum	99.70
Mean	98.25
Std. Deviation	1.442
Std. Error	0.645
Lower 95% CI of mean	96.46
Upper 95% CI of mean	100.0

- - - - - - - - - 2.5 SD threshold

Supplementary Figure 3.

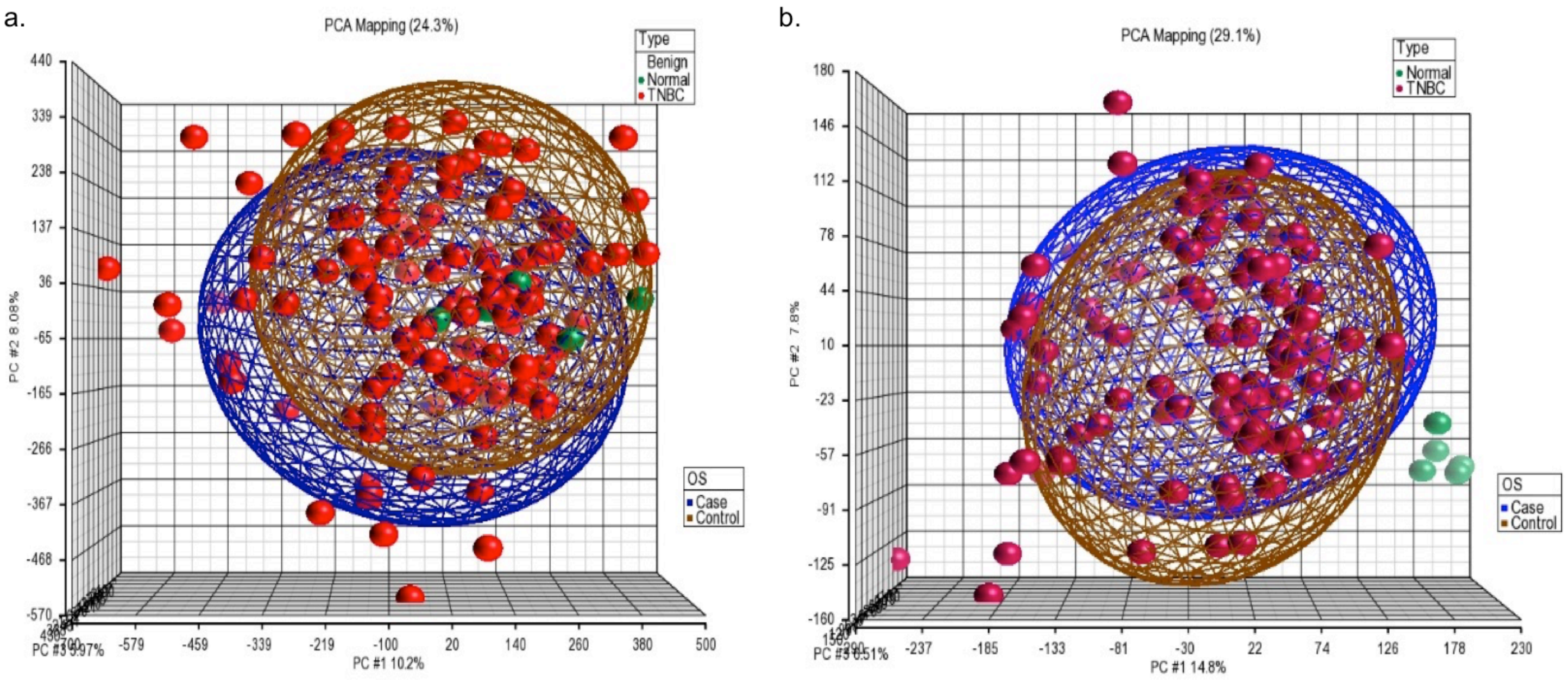

Supplementary Figure 4.

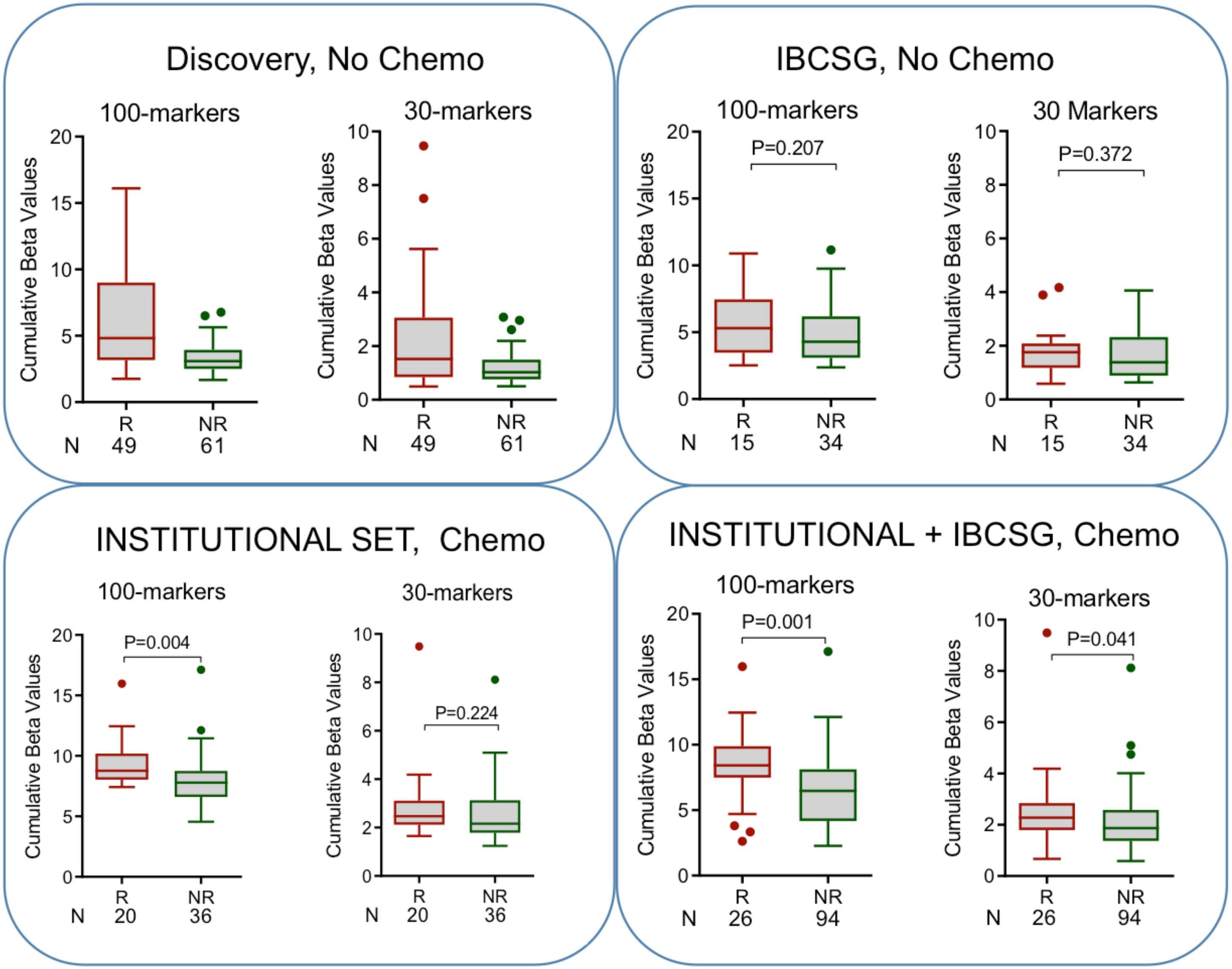

Supplementary Figure 5.

Cross- Platform Validation of the Methylation Array – IBCSG No Chemo samples (12 recurrences; 30 controls)

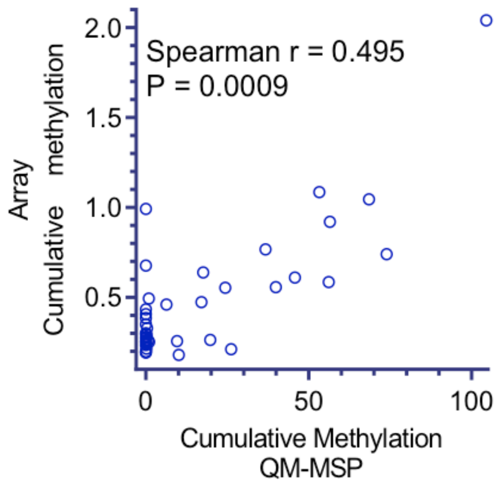

Target ID	SYMBOL
cg20071051	NEK9
cg08193650	ZFP36L2
cg12589600	GTF2F1
cg14237711	ZSCAN5A
cg18191418	Not assigned
cg26029997	TREX1
cg01951637	CBFA2T3
cg11070056	PRMT6
cg22884792	WNT1

Supplementary Table 1.

Table S1 100 CpG Recurrence Markers Selected in the Discovery Cohort

TargetID	SYMBOL	Ratio Mean β R/NR	R mean β-value*	NR mean β-value*	CpG Island	REGULATORY FEATURE	CHR	GENOMIC LOCATION	In 30 Marker Set	GENOMIC SOURCESEQ
cg01000656	SPATA1	2.648	0.032	0.012	Island	Promoter	1	84971486		AGCAGGCGCCGCGTGGGTCTACGCTGTGTTTTAGGGGGACGATTAGTTCG
cg17750334	CENPF	2.147	0.028	0.013	Island	Promoter	1	214776613		CGCGCCAGAACTGTACTCTCCGAGAGGTCGTTTTCCCGTCCCCGAGAGCA
cg25277534	DDAH1	2.000	0.043	0.021	Island	Unclassified	1	85930476		CGCACACCACGGCCACGTCCTCCACGAAGACGCAGTCCGGAAGGCTCTCG
cg18159850	OBSCN	1.839	0.029	0.016	Island	Promoter	1	228401278		GTCCACTGAGGCCGCGTGGTTCAAGGAGGAGACGCGGTTGTGGGCGAGCG
cg11070056	PRMT6	1.738	0.074	0.042	Island	Unclassified	1	107600091	Yes	TCCAGCTCCTGCTCCAAGCCGGCGCGGGAGAGCTCTAGCTGAGCAAAGCG
cg07914309	RGS2	1.706	0.026	0.015	Island	Promoter	1	192778123		TTGCGGCTGGGCGTGCGTCGCAGCATTTATGAGGCCTCGGCTTGGGGGCG
cg13904493	KIAA1804	1.692	0.037	0.022	Island	Unclassified	1	233464194	Yes	CGCGGTACACCTGCCCGAAGCCCCCAGCGCCGATGAGCTCCTTCAGCTCC
cg09897416	HIST2H2AB	1.677	0.034	0.020	Island	Promoter	1	149859252		CGGGACAACAAGAAGACGCGCATCATCCCTCGCCATCTGCAACTAGCCGT
cg19769760	POU3F1	1.656	0.075	0.046	Island	Unclassified	1	38511105		GGGTCACTGCACTGAGCCGGGCAGTGTGTGGTGGTGGTGGTGGTGCAGCG
cg17079378	RAD54L	1.640	0.045	0.028	Not assigned	Promoter	1	46713440		TTCGATTGACCCGGTCTTGGCGGGTCGGTGAGTCTTGGCGGCTGTTAACG
cg04458869	LASS6	3.011	0.039	0.013	Island	Promoter	2	169312687		GCCCTGCGGGTGTGGGCGAGTTGGCCGCGTGTGTGCCTCGCTGTTTGACG
cg08193650	ZFP36L2	2.567	0.093	0.036	Island	Unclassified	2	43451868	Yes	CGCCAACAACGCCTTCGCCTTCGGTCCGGAGCTCAGCAGCCTCATCACGC
cg08535260	Not assigned	2.302	0.055	0.024	Island	Unclassified	2	127643971		CGGGCCCATCACCCAGAGCCCCAAACGCACCCTCGCGGCGCTTCAGCGGG
cg00878630	COPS7B	1.944	0.048	0.025	Island	Promoter	2	232651109		GGTGGAACCTTGTGTGGGCGGAACGCGAGTGCAGCGGTGGGAGGCTTCCG
cg01987515	COPS7B	1.936	0.051	0.026	Island	Promoter	2	232651086		CGCGTTCCGCCCACACAAGGTTCCACCCGCCGCGTTTACAGCCTTCGACC
cg02943303	EIF5B	1.765	0.099	0.056	Island	Promoter	2	99952887		ACCTTGGGTTCCGGGCTGCTGGGCGGGAGGCGCACGCGCAGAACTGTTCG
cg11162249	NUDT16	1.941	0.042	0.022	Island	Promoter	3	131100713	Yes	CGCATGACGCCAGCCCGACCCCAGCGCCAGGGCCTCGCCTAGCTCCAGCC
cg18191418	Not assigned	1.833	0.095	0.052	Island	Promoter	3	128336579	Yes	AACACTGCGGCACGTCACGAGGCGGGCACCGGGGAGTATGGGCGAAATCG
cg26029997	TREX1	1.801	0.072	0.040	Not assigned	Promoter	3	48507610	Yes	CGGGCCCACGCCAAGTTTCACTTCCCGCCACTGCTGCCAGCGAGAGCCGC
cg17285536	OGG1	1.725	0.043	0.025	Island	Promoter	3	9791531		CGCCTCAGAACGGCCCGCCCACCCTGATTTCTCATTGGCGCCTCCTACCT
cg02510164	MCF2L2	1.703	0.046	0.027	Island	Promoter	3	183146421		CTTACTCGGAACCGTGGGGCGGGGAGACAGTGACTGAGGCGCTCCAGTCG
cg05585810	RAP2B	1.699	0.039	0.023	Island	Promoter	3	152880101		CGCGCAATTAGCAGCCACCTCCGCAGCCCGCCGCCACCGCCTCCCTGCCC
cg24623434	RPP14	1.632	0.060	0.037	Island	Promoter	3	58291823		AGCCGTGTGTGCAATTTGCTTGGGGTGCGGTTTAGGGCGGGTTTTTTCCG
cg22958231	SPATA4	1.645	0.054	0.033	Island	Promoter	4	177116835		GCCCCGCGGTCCAAAATCTGAGTAAAGCGGAAGTACGGGCGGCTGCTGCG
cg20007130	DCHS2	1.634	0.056	0.034	Island	Unclassified	4	155413075		AGGCAGGCGGCGGAGGCTGGGTCGGAGCAGGAGTCGCGCGGTCCCTGGCG
cg23429847	ISOC1	1.632	0.037	0.023	Island	Promoter	5	128430259		CCCCGGAGGGCGGGCCGCGGCGTAAGGGTGGGTGGTGGGGCCGGGGTGCG
cg25211525	C6orf145	2.012	0.034	0.017	Island	Unclassified	6	3751739		CGTGCCCTCAAACACCGCCGAGGCCATGTCGCACGCATGCCCCCGCCAAG
cg11927033	PKIB	1.930	0.038	0.020	Island	Promoter	6	122793073		GCCTGGGTAGGATAAAACACGGAGCGCGCAGCGCTGAGACTGCGCATGCG
cg18892128	Not assigned	1.831	0.087	0.047	Not assigned	Unclassified	6	31409575	Yes	GCCTGGTCCCCGAAAGCGCTCAGTCAGAATCCGAGAGCACTGTGGCGGCG
cg03300581	CNR1	1.831	0.037	0.020	Island	Unclassified	6	88875844		GAGTGGGGTGTGCCTCCGGCGGGCGGTCAGCAAGTCAGTCCGTCCGAGCG
cg06512858	SOX4	1.799	0.044	0.025	Island	Promoter	6	21596796		AAGGCTCCGGGGTGACGAATTTGGCCGATGGCAGATGTTTTGGGGGAACG
cg19861329	FOXO3	1.789	0.045	0.025	Island	Unclassified	6	108881880		GCCGCGGTCAGAGCCCGAGCTCGGGTGTGCGTGCGTTTGTTTATGTTTCG
cg18418460	ULBP3	1.656	0.052	0.031	Island	Unclassified	6	150390014		AAAGAGCAGCGAATCGGAACTGAGCGTGGGGGCAGTCCGGGGAGATCGCG
cg15816080	TPST1	2.032	0.030	0.015	Island	Promoter	7	65670215		ACATCTGCTACGATTGGCTGAGCGGACGATGTGTCGGCTGCTCTGATTCG
cg23755113	TPST1	1.807	0.046	0.026	Island	Promoter	7	65670237		CGTCCGCTCAGCCAATCGTAGCAGATGTGTGATCCCGCCACCTCCCCGGA
cg22813794	STYXL1	1.767	0.153	0.087	Island	Promoter	7	75677469		GGAGAGCAGCGCTGGCAGGCCGGGCGAGGGCGGAGAGCATGGCTGGAGCG
cg24133836	AASS	1.677	0.085	0.051	Island	Unclassified	7	121784386	Yes	CGCCCTGAGGGAACAACCTTCTCATCTGCCAATCGGCGGCGCTGCCAGTT
cg03184037	WRN	2.246	0.109	0.048	Not assigned	Unclassified	8	30891338		TGGAGGGGCGGGTCGGGTAGGTCTCCCGGAGCTGATGTGTACTGTGTGCG
cg10594709	Not assigned	1.988	0.108	0.054	Island	Unclassified	8	144489858	Yes	GCAGCGGACGTGGGGGGTAGGAGCGCGCAGGGCGGGGGCGTCAGATGGCG
cg02196694	TOX	1.692	0.126	0.075	Island	Unclassified	8	60031540	Yes	GAGCGTCGGGCGCAGCGGCGGGCTGGGCTGGAGGTGGATAAAATCTTACG
cg01556929	VPS28	1.689	0.033	0.019	Island	Promoter	8	145654081		GGCGGGTGGGCGGGGTAAGACCGGGCTTCGAGTCTGCTGCGCCGGTTACG
cg25336579	PSAT1	1.883	0.037	0.019	Island	Unclassified	9	80912260		CGCAGGGATGCAAACCCACCCGCGTGCGTGCTCCCGCCTGAACCTCACTC
cg18435900	ZNF485	2.499	0.027	0.011	Island	Unclassified	10	44101962		CGCCCAGAGAGTCAGCGACCACACCACGCCGTTCGGACAGTAACCACCAG
cg10107304	C10orf26	1.923	0.111	0.058	Not assigned	Promoter	10	104503664		GAAGCGCTGGAACTGGAGAGTCTCTCGCGTGTCTGGGAGGGTGTTTTGCG
cg05739190	CCNJ	1.675	0.052	0.031	Island	Promoter	10	97803386		TTAACAATGGGCGGCGCGAGCGTCCAGGCGCTGGGCTCTAGCTGAGGCCG
cg18977061	PTPLA	1.625	0.038	0.024	Island	Promoter	10	17659544		CGCTCCCGAGGAAAGTGCACCCGCCTCGCTAGCTGCGGGCGCCTCTTCAT
cg19948397	C11orf61	2.363	0.032	0.013	Island	Unclassified	11	124670177		CGGAAATCCATCGCTGTCCGACCCTTCCACGCCTCGGGGTGCCTCCCCGC
cg09602577	C11orf61	1.962	0.037	0.019	Island	Unclassified	11	124669905		AGACGAACGCGCTCATCGCAGTGTGGGGCAACGAGCGGCTGGTGGAGGCG
cg26583055	C11orf61	1.780	0.047	0.027	Island	Promoter	11	124670419		CGCCCGGCAGCCACATCCTCAGCAGCAGAGACCCGGAGCCATCCGCCCGC
cg04767207	VPS37C	1.779	0.069	0.039	Island	Promoter	11	60929031		GCGGCCGCGAGCGTGTGTGTGTGGCACGATGTCGTGGTAACTCAGAGGCG
cg19899312	Not assigned	1.755	0.078	0.044	Island	Unclassified	11	76757278	Yes	TGTGTGCGGGATGCGGGGTGCGGGGTGCGGGGCGTGCCCCCTGGCGGCCG
cg01885963	ZNF215	1.706	0.131	0.077	Island	Promoter	11	6947628		CACCGTGAGTCCATTCACAGGGCCAGAGTTCCAAGTGTGCGGCGTGGGCG
cg24449148	TMEM216	1.642	0.066	0.040	Island	Unclassified	11	61160132		CGGCGCCATCTTCAGTCCCCGTGGCAGCATACGCTGCCACAGCGGCTCCC
cg01703419	Not assigned	2.437	0.051	0.021	Island	Unclassified	12	108297587	Yes	CTGGGCGGGCGGCCAACGTGGCTGGGCGTGGCTGGGCGTGGATGGGCGCG
cg24677744	Not assigned	2.038	0.051	0.025	N_Shore	Unclassified	12	29302016		AGTGGATCCGCTGGTGCTGAACGAATGGAAGCGCGGGGCGGGCCTGCGCG
cg14262439	TMEM132B	2.034	0.120	0.059	Island	Unclassified	12	126018155	Yes	CGCCCACGAGGTCCTTCCCCGGAGGAGAGGCGGATGCCAATCAAAATGCC
cg22884792	WNT1	1.716	0.051	0.030	Island	Unclassified	12	49371987	Yes	CGCCCGCCTCACTCAGCTGAGCGTCCGGAGCCCGTCGAGGACTAGCATCC
cg10360769	LOC338799	1.715	0.055	0.032	N_Shore	Promoter	12	122240872		CGCGGCAGCCAATCAGCGCGCGGCTTCTATAGGGCTTGAGTTATTAGACG
cg26197220	TSC22D1	2.070	0.035	0.017	Island	Unclassified	13	45150229		CGCCCCCGGTACCCAACACAATGCACCAGCCGCCTGAGTCCACCGCCGCG
cg15608786	SLAIN1	1.682	0.044	0.026	Island	Unclassified	13	78271880		CGGCCGCGGCAGCCACCACCGAGCTGATCTGCACATGCTCAGTGCGTCCC
cg20071051	NEK9	2.611	0.080	0.031	Island	Promoter	14	75593943	Yes	CGGGAACAAGGGCATCACTGACGGTACGGCCTCTCGGGTCTATTGACCCT
cg08847533	NEK9	2.561	0.072	0.028	Island	Promoter	14	75593920	Yes	CTGCAAAGTGAGGCCTCGGGCTAGGGTTGGCGGGCGCGTGGTGGGAACCG
cg09944325	NEK9	2.159	0.109	0.051	Island	Promoter	14	75593969		GCGTGGAACCGAAGGGCTCCCACGGGAACAAGGGCATCACTGACGGTACG
cg01141940	RTN1	1.850	0.120	0.065	Island	Unclassified	14	60097247	Yes	CGGCGAGCCCCCAAAGCCCGCACGATGCAGGCCACTGCCGATTCCACCAA
cg01992424	NEK9	1.713	0.116	0.068	Island	Promoter	14	75593964		GAACCGCGTGGAACCGAAGGGCTCCCACGGGAACAAGGGCATCACTGACG
cg10174687	FAM98B	3.222	0.081	0.025	Island	Promoter	15	38746780		TGGGCTAGCTCGCGCCCGCAGGTGATATTTGTGTTGACGTAGTTCTGGCG
cg21595526	GABPB1	2.449	0.036	0.015	Island	Unclassified	15	50647267		CGGCCCCGCTACACACAAAGCGCTTCAGCTGCACAGGGCGCTATTTTCCG
cg03355298	B2M	2.216	0.044	0.020	Island	Promoter	15	45003704		CGCGACGCCTCCACTTATATTAAACGCGTGCCCAGCCAATCAGGACAAGG
cg06917634	PSMA4	1.755	0.041	0.023	Island	Unclassified	15	78832804		ACCCAGCGGTTCTGCGCATGCGCGGGGGCCATATTAGCAGCGGTTATTCG
cg06842781	NDNL2	1.715	0.019	0.011	Island	Promoter	15	29562077		GTGCTGCGTCATGAAGCCTGCGCCTTGCGTCAGGGCGGCTGGGCGGTGCG
cg24451981	BUB1B	1.706	0.063	0.037	Island	Promoter	15	40453319		TGGGCTTGAGGTGGCCGGTTTGTTAGGGAGTCGTGTACGTGCCTTGGTCG
cg15671705	ALDH1A3	1.682	0.036	0.021	Island	Unclassified	15	101419479		GCTCGGGCAGGGTGGGGCGCAGCGGGCGTCTGTCCTGCTAGCGGGGATCG
cg01951637	CBFA2T3	1.798	0.088	0.049	Island	Unclassified	16	89007466	Yes	GGACCAAGCTGGAAGGAGCAGCGACTCCCGGACCGAGTCGCGAGTGTGCG
cg08547343	CENPBD1	1.685	0.058	0.034	Island	Promoter	16	90038815		CGCCTTTAGACTTATCTTAAATCGGGCATCGGCATCAACACTTATTTCTG
cg09876101	WDR90	1.668	0.031	0.019	Island	Promoter	16	699357	Yes	GTAATGGCGGAAGTGGCACCGTTGCCAGGCAGCCGTTGCCTGGCGTCGCG
cg02727363	EXOSC6	1.655	0.053	0.032	Island	Promoter	16	70284928		TTCCAGGAATTCTCGACGCAAACTGGAGGCCGATGGCGCGGGTCTTTTCG
cg00688900	GGNBP2	2.212	0.041	0.018	Island	Promoter	17	34901364		CGCTGGGCGCTGATTGGCTGAGCGTGTGTGGAATCGGGTGATGGGAAACG
cg02392688	GIT1	2.159	0.041	0.019	Island	Unclassified	17	27916351		GAGGATGTCCCGAAAGGGGCCGCGAGCGGAGGTGTGTGCGGACTGCAGCG
cg14888846	CCDC55	1.704	0.072	0.042	Island	Promoter	17	28443962		GGATGAGAAACTACGGCGACTGTATCTTTGGCCGAGGAGTTTTAAATGCG
cg18658096	AARSD1	1.685	0.054	0.032	Island	Promoter	17	41116483		TCACGCTGACACCAGAACGCCATACCTGCAGGCGTGTGAGGAGGCGCACG
cg00319395	RPAIN	1.661	0.037	0.022	Island	Promoter	17	5323036		CGAGCTTCCGGGCCGTCTGCTCGGTGATTGGCCGAAACAGTGAGTGGACG
cg26708638	ZNF444	2.351	0.038	0.016	Island	Promoter	19	56652447	Yes	CGCCAACACTGGAACTACATTTCCCAAGATACGCGAAAGCGTCGTCTGCA
cg17319787	Not assigned	2.251	0.048	0.021	Island	Unclassified	19	9420361	Yes	CCTGCCCCGGGTCGGGTCCGCGGGAGACCGAGGGCTGGGTTTGGGAATCG
cg12589600	GTF2F1	2.092	0.047	0.022	Island	Promoter	19	6393177	Yes	TCGCTTCCGGGTTAGGCGGCCGCAGAAGCGTAGACCGAGTAGCTTGAGCG
cg14237711	Not assigned	1.933	0.079	0.041	Island	Promoter	19	56826044	Yes	GACCGTCACTAGTAGACGTTTCTGAGTGTGTCGCGGTCACCATGGTAACG
cg04541675	FAM71E1	1.929	0.088	0.046	Island	Promoter	19	50979561	Yes	GCGGGACTACGCGCACTGTGACGTCATGATGCGGAGCGGGCTTTATGACG
cg23307203	GDF15	1.854	0.052	0.028	Island	Unclassified	19	18499309		CGCAGTCGGACCAACTGCTGGCAGAATCTTCGTCCGCACGGCCCCAGCTG
cg02837212	ETFB	1.835	0.093	0.051	Island	Promoter	19	51869672	Yes	CGCCTGCGCGCAGCACGGTCAGCCAGTCAACCGCTGCAGAGCCCGCCTCC
cg21877656	ZNF329	1.819	0.050	0.027	Island	Promoter	19	58662188	Yes	CGCGCACATCCTTGTCCCGGCAGCCGCGCAAGCGCAATCGCCCGGAACAT
cg22286978	A1BG	1.770	0.039	0.022	Island	Promoter	19	58858806	Yes	CGCCGGCAACTACAGGTGCCGCTACCGCTCCTGGGTGCCCCACACCTTCG
cg06867065	CLDND2	1.745	0.131	0.075	Island	Promoter	19	51871233		AGCCACGCTGGCGGTGACTGTGGCGTGCATGGTGCTGGCGGTGGGTGTCG
cg02951558	FAM71E1	1.705	0.091	0.054	Island	Promoter	19	50979574		CACTGTGACGTCATGATGCGGAGCGGGCTTTATGACGTCACACAAGAGCG
cg19429286	Not assigned	1.671	0.051	0.030	Island	Unclassified	19	9420514	Yes	CAGACGCGGGCGGAGGCGTAGGGTGACAAGGCTGTCGACTCGCGCATGCG
cg02666184	ETFB	1.653	0.147	0.089	Island	Promoter	19	51869680	Yes	TCTGTCGGGGAGGCGGGCTCTGCAGCGGTTGACTGGCTGACCGTGCTGCG
cg14602640	CGB7	1.635	0.053	0.033	Island	Unclassified	19	49559588		GCTTACAGCGGCCTGAGCGGGAGTTCTCGGTGCTGTAGGCTTTCGGGACG
cg14942918	ONECUT3	1.633	0.063	0.039	Island	Unclassified	19	1757833	Yes	CGCCCCAACGCTGGTGCTCGGAAAGGGAACTCTGCCCCCGCCCCGCCCCC
cg21803754	PSMA7	2.335	0.033	0.014	Island	Promoter	20	60718392		CCGGGAGTCGCGGGTGCGCGCGGGCCGTGAGTGTGCGCTTTTGAGAGTCG
cg14096051	THAP7	2.282	0.033	0.014	Island	Unclassified	22	21356040		GCATGCGCTAGTTGTTGCTCGCCTCGGGTCGCAACTGCGTAGAGGCTTCG
cg20786683	SMTN	1.885	0.030	0.016	Island	Promoter	22	31477247		CGCGCCAGCCAACCCTCGATTGGGTACCTCCCACTCTCGTGCCCCATTGG
cg05454323	RRP7B	1.634	0.049	0.030	Island	Promoter	22	42978119		TGGCGCGCTGATTTGCCTCTGAGCGGATGAAGGTTGAGCCATGGCGGGCG

* Mean ave.beta value

Supplementary Table 2.

Table S2 Chromosomal Localization of Methylated CpG Loci and in the Combined Chemo Group*

TargetID	SYMBOL	Log. Reg. P-Value	Chr. Band
cg17319787	ZNF699	*0.0003*	19p13.2
cg01703419	Not assigned	*0.002*	12q23.3
cg02196694	TOX	*0.002*	8q12.1
cg04541675	FAM71E1	*0.002*	19q13.33
cg09876101	WDR90	*0.002*	16p13.3
cg14942918	ONECUT3	*0.002*	19p13.3
cg20071051	NEK9	*0.002*	14q24.3
cg22286978	A1BG	*0.002*	19q13.43
cg26708638	ZNF444	*0.002*	19q13.43
cg11162249	NUDT16	*0.008*	3q22.1
cg13904493	MAP3K21	*0.008*	1q42.2
cg14237711	ZSCAN5A	*0.008*	19q13.43
cg24133836	AASS	*0.008*	7q31.32
cg01141940	RTN1	*0.027*	14q23.1
cg02837212	ETFB	*0.077*	19q13.41
cg14262439	TMEM132B	*0.077*	12q24.32
cg21877656	ZNF329	*0.077*	19q13.43
cg02666184	ETFB	*0.187*	19q13.41
cg08193650	ZFP36L2	*0.187*	2p21
cg12589600	GTF2F1	*0.187*	19p13.3
cg10594709	Not assigned	*0.380*	8q24.3
cg11070056	PRMT6	*0.380*	1p13.3
cg22884792	WNT1	*0.380*	12q13.12
cg18191418	Not assigned	*0.661*	3q21.3
cg19429286	ZNF699	*0.661*	19p13.2
cg19899312	Not assigned	*0.661*	11q13.5
cg01951637	CBFA2T3	*1.000*	16q24.3
cg08847533	NEK9	*1.000*	14q24.3
cg18892128	Not assigned	*1.000*	6p21.33
cg26029997	TREX1	*1.000*	3p21.31

* Logistic regression p-value is based on median methylation for each individual CpG locus of the 30 marker set in the combined IBCSG + Institutional Chemo cohort (26 cases and 94 controls). Correction for multiple testing was not performed.

Supplementary Table 3.

Table S3 Enrichment of CpG Loci Located on Chromosome 19		
**Marker Sets**	**# Situated on Chromosome 19**	**% (Enrichment)**
Total array - 485577 loci	25521/485577	5.26 % probes specific for Ch. 19
100 locus recurrence marker set	15/100	15% (2.85 fold)
30 locus recurrence marker set	11/30	36.7% (6.98 fold)
14 loci of 30 marker set with signifcant association between recurrence and methylation *	6/14	42.9% (8.12 fold)

* Determined by by logistic regression, based on the median methylation level
